# Supplementary material for: An unexpected strategy to alleviate hypoxia limitation of photodynamic therapy by biotinylation of photosensitizers
Source: Nat Commun. 2022 Apr 25;13:2225. doi: 10.1038/s41467-022-29862-9 (PMC9038921; doi:10.1038/s41467-022-29862-9)
Supplement: Supplementary file 1 — Supplementary information [file 41467_2022_29862_MOESM1_ESM.docx]

Supplementary Information

An Unexpected Strategy to Alleviate Hypoxia Limitation of Photodynamic Therapy by Biotination of Photosensitizers

Jing An^1^, Shanliang Tang^1^, Gaobo Hong^1^, Wenlong Chen^1^, Miaomiao Chen^1^, Jitao Song^2^, Zhiliang Li^2^, Xiaojun Peng^1^, Fengling Song*****^,1,2^, and Wen-Heng Zheng*****^, 3^

^1^State Key Laboratory of Fine Chemicals, Dalian University of Technology, Dalian, 116024, China

^2^Institute of Molecular Sciences and Engineering, Institute of Frontier and Interdisciplinary Science, Shandong University, Qingdao, 266237, China

^3^Department of Interventional Therapy, Cancer Hospital of Dalian University of Technology, Liaoning Cancer Hospital and Institute, Shenyang 110042, China

*KEYWORDS photodynamic therapy • photosensitizers • tumor hypoxia • biotin • white light*

*Correspondence authors email: [songfl@dlut.edu.cn](mailto:songfl@dlut.edu.cn); [songfl@sdu.edu.cn](mailto:songfl@sdu.edu.cn); Mir2yue2@163.com

**Supplementary Figure 1**. Synthesis of compound **1-11**.

**Mass and NMR spectra of compounds**


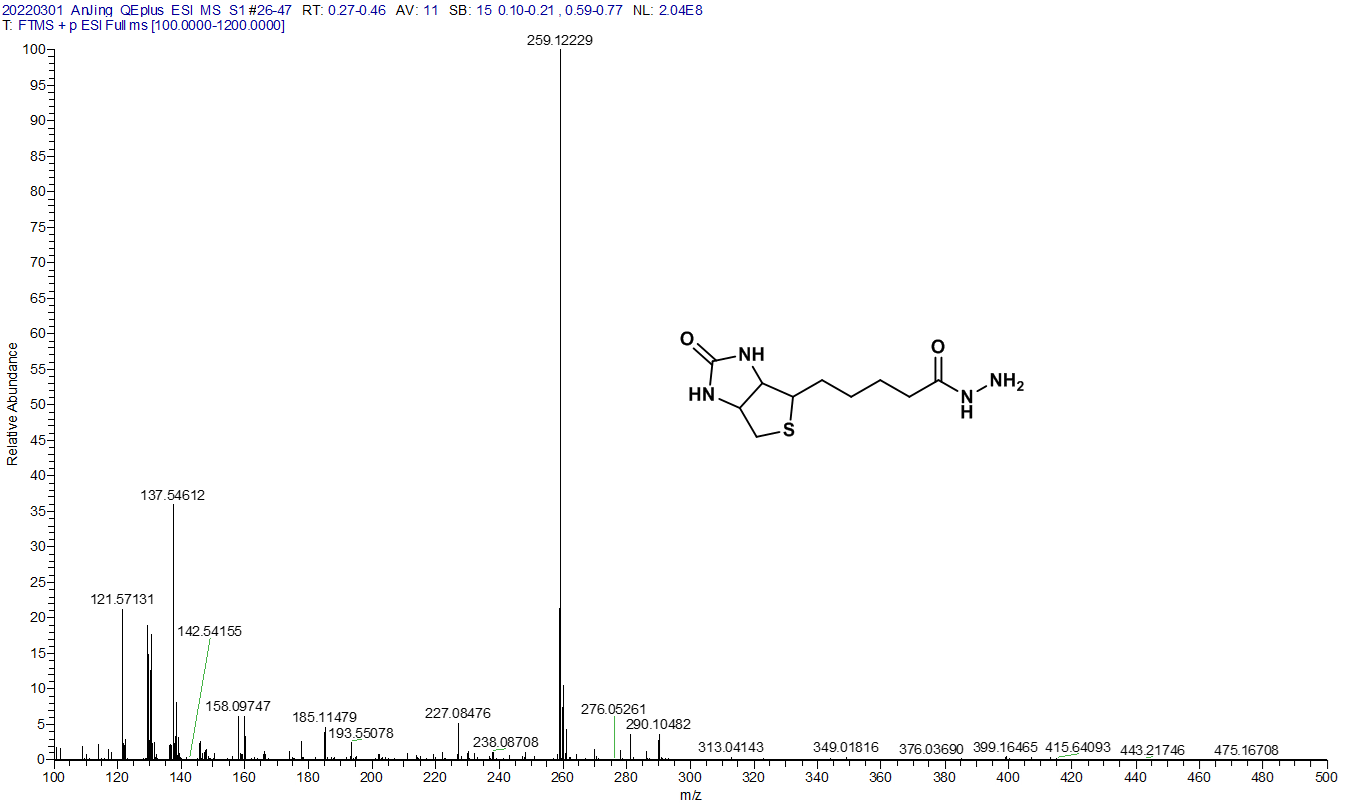


**Supplementary Figure 2.** HRMS of compound **11**.


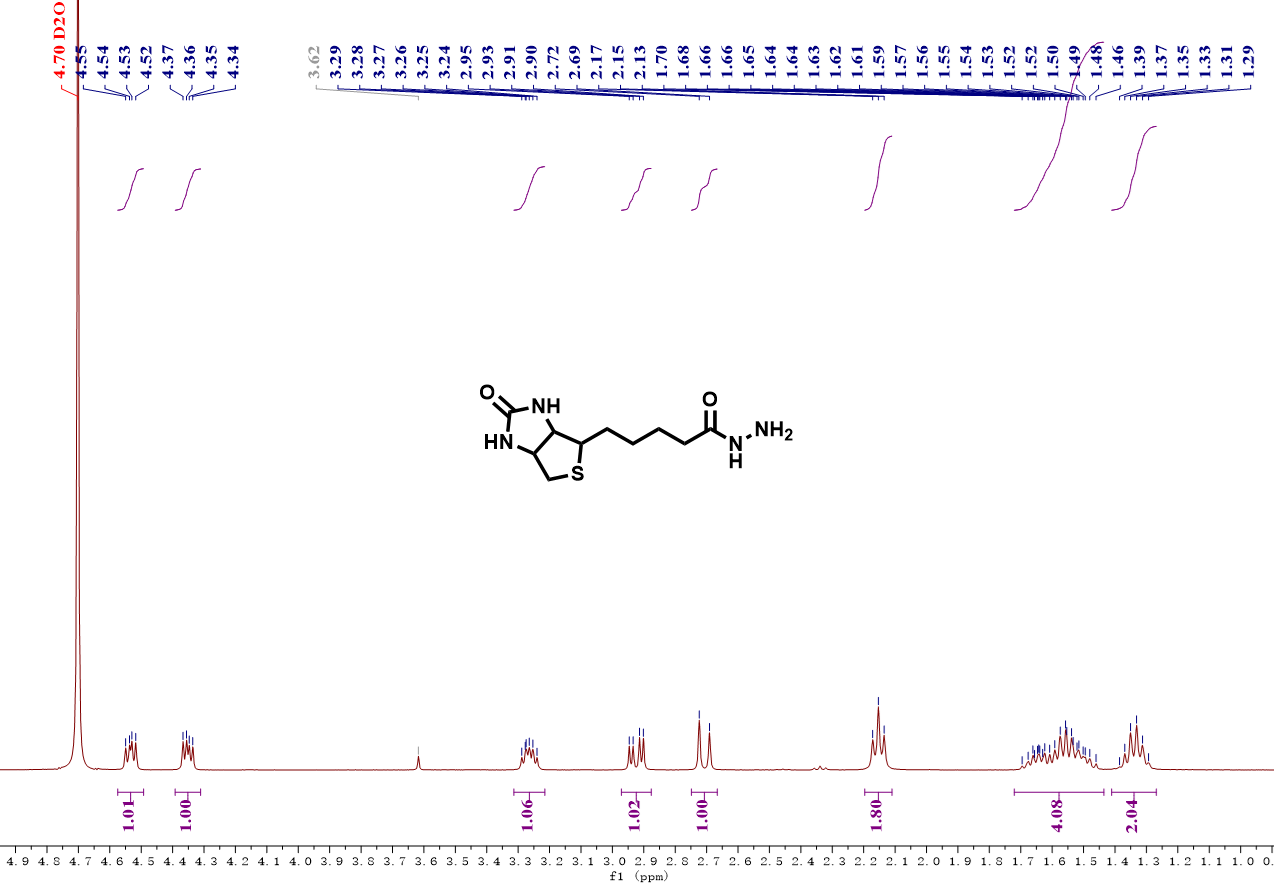


**Supplementary Figure 3.** ^1^H-NMR spectrum of compound **11** in D_2_O.


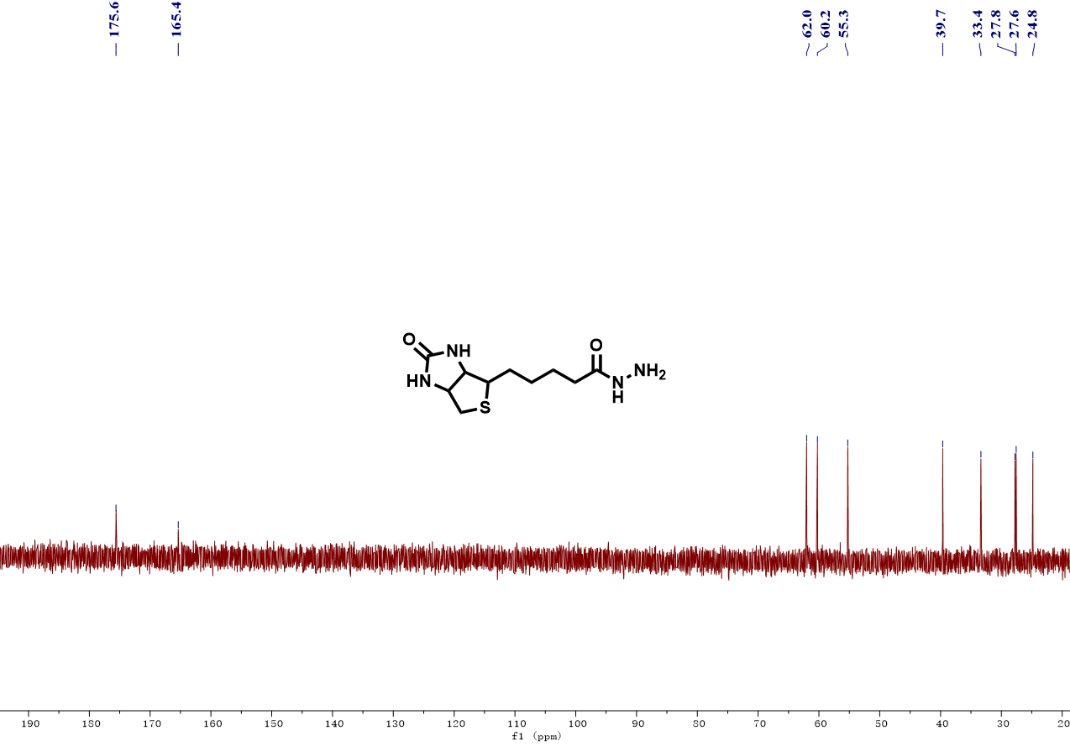


**Supplementary Figure 4.** ^13^C-NMR spectrum of compound **11** in D_2_O


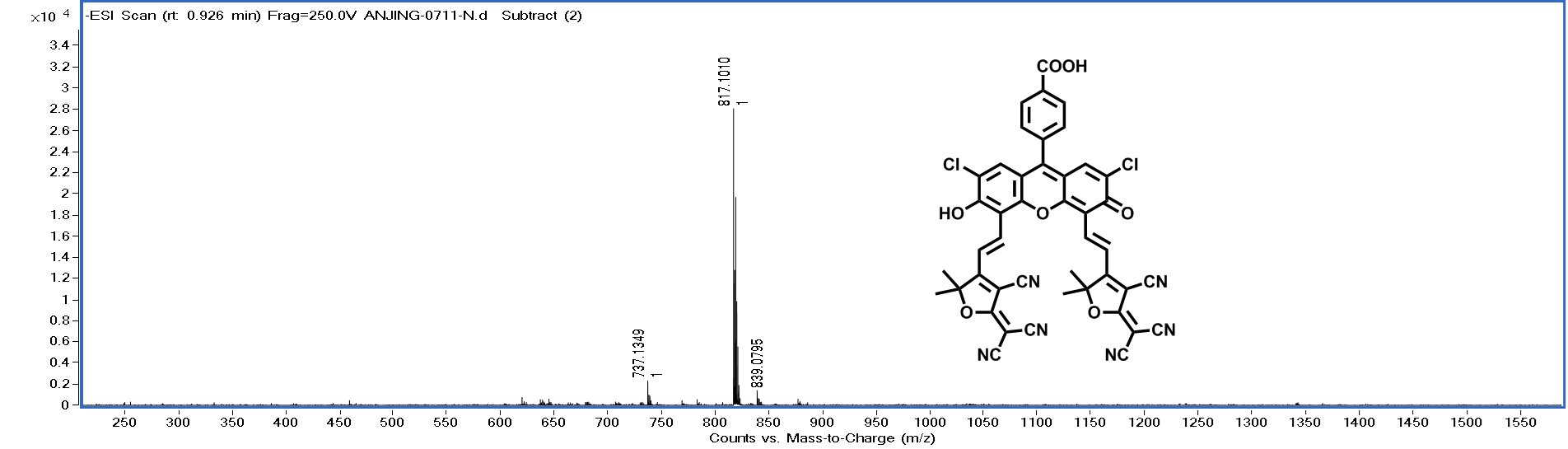


**Supplementary Figure 5.** HRMS of compound **4**.


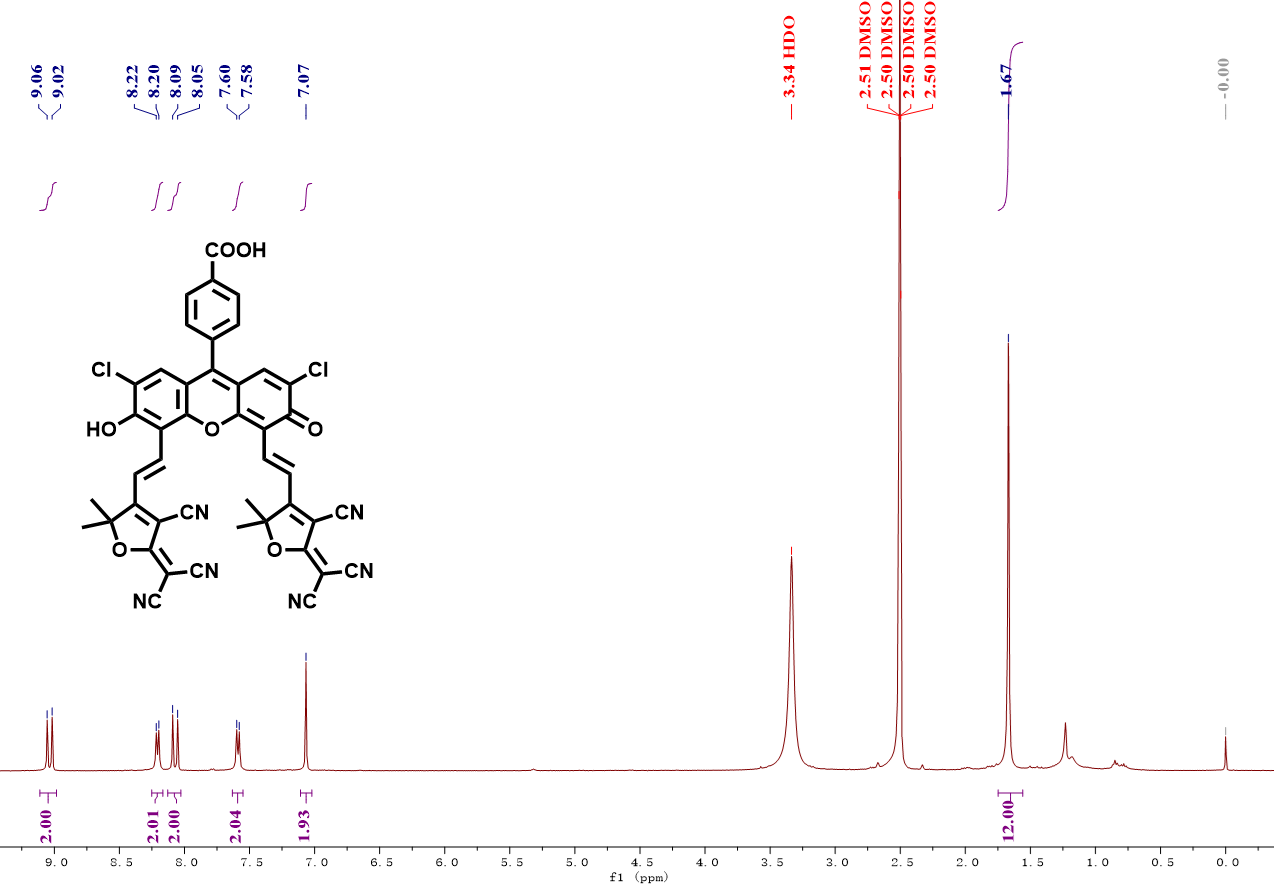


**Supplementary Figure 6.** ^1^H-NMR spectrum of compound **4** in (CD_3_)_2_SO.


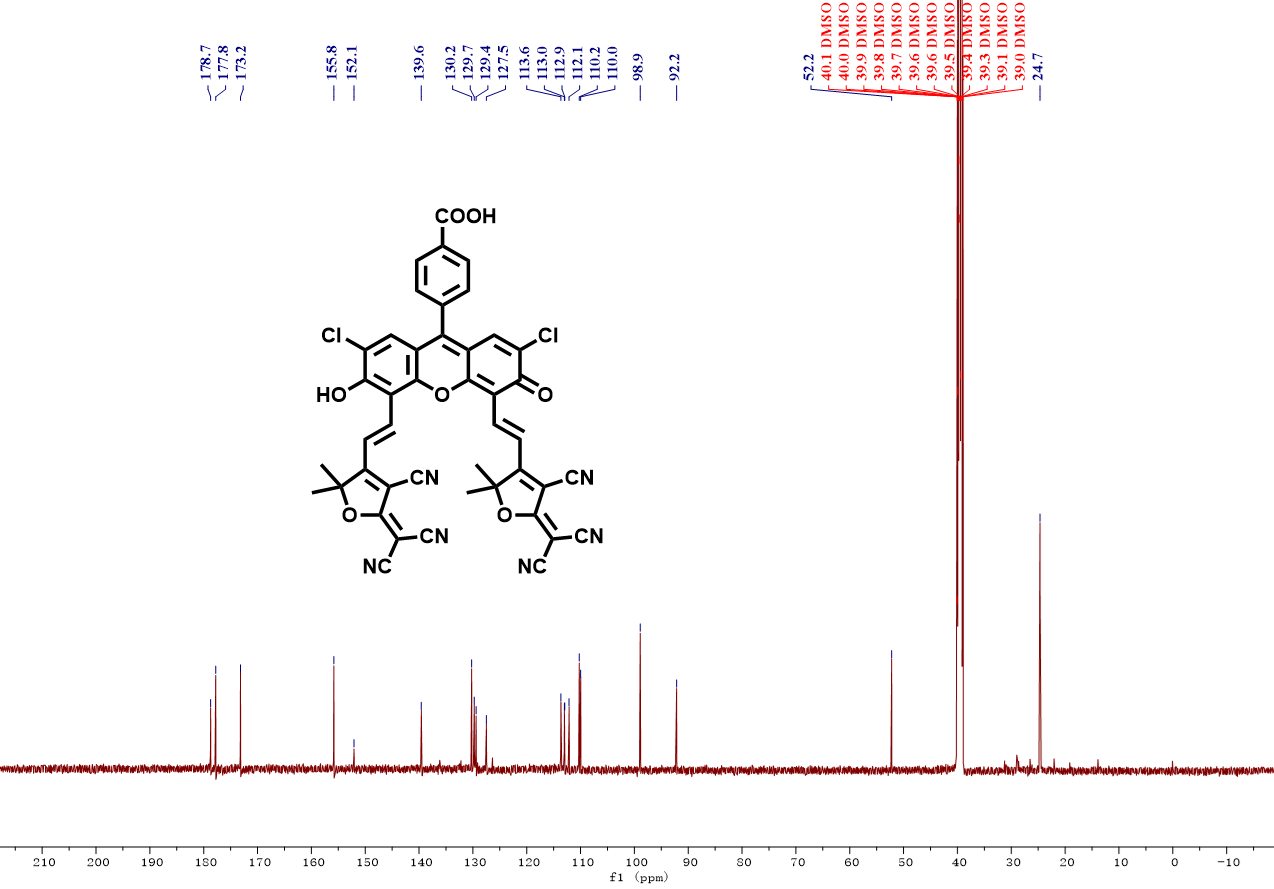


**Supplementary Figure 7.** ^13^C-NMR spectrum of compound **4** in (CD_3_)_2_SO.


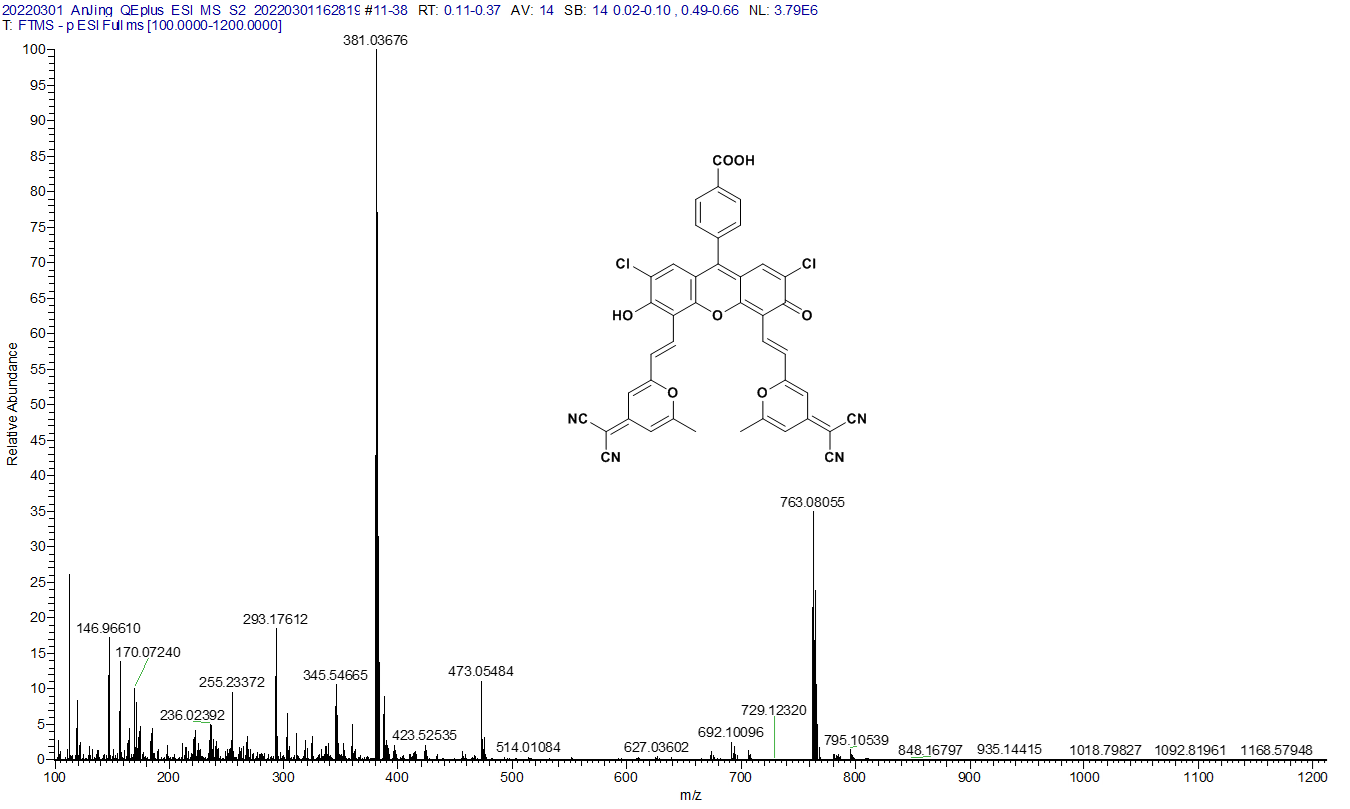


**Supplementary Figure 8.** HRMS of compound **4**.


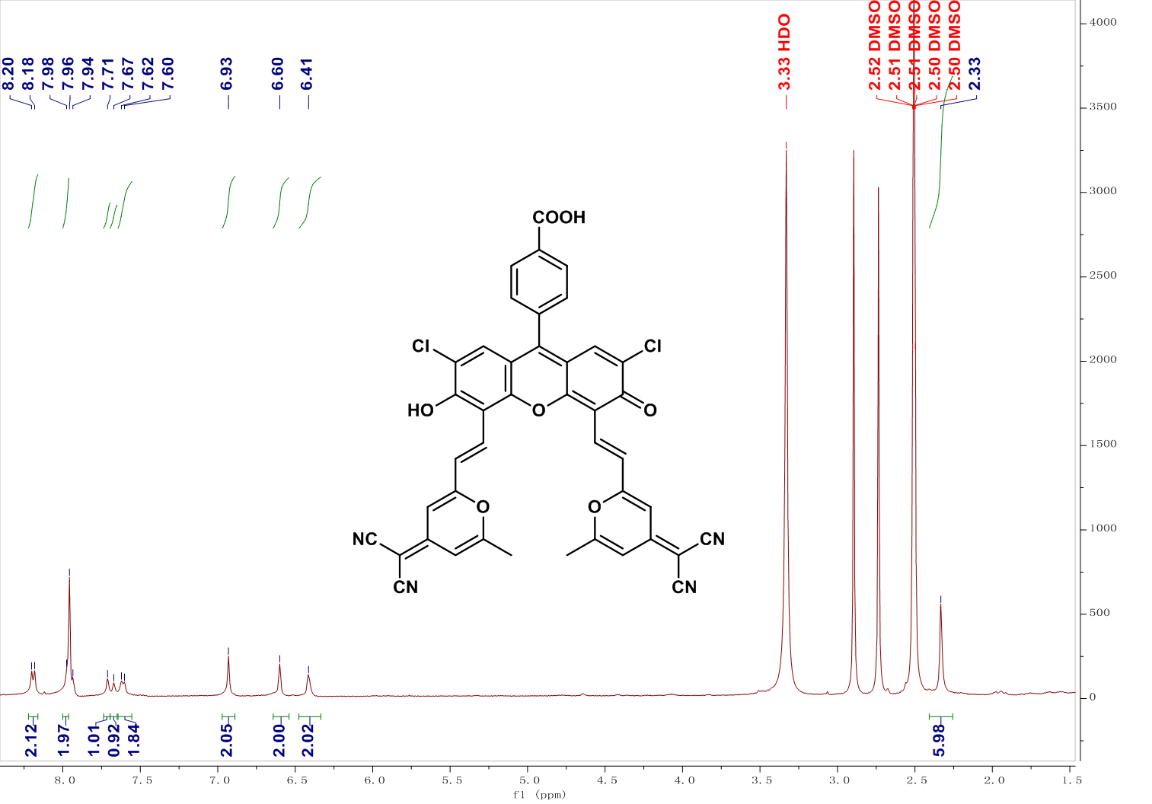


**Supplementary Figure 9.** ^1^H-NMR spectrum of compound **5** in (CD_3_)_2_SO.


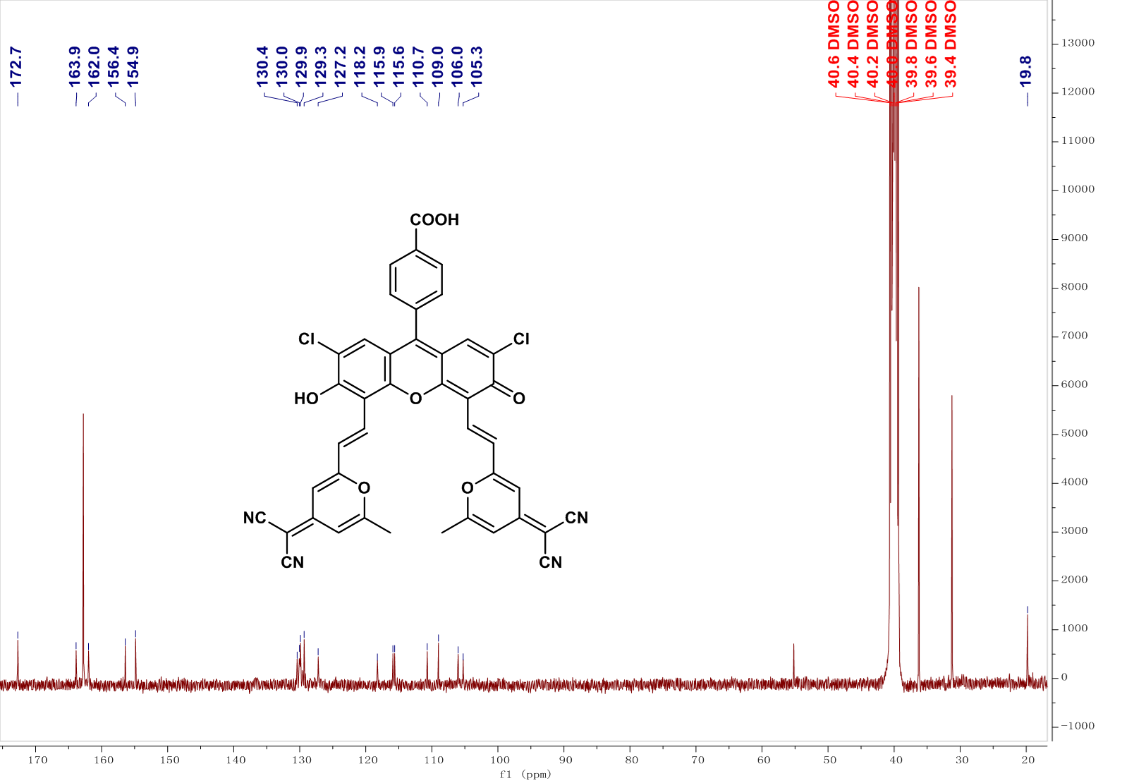


**Supplementary Figure 10.** ^13^C-NMR spectrum of compound **5** in (CD_3_)_2_SO.


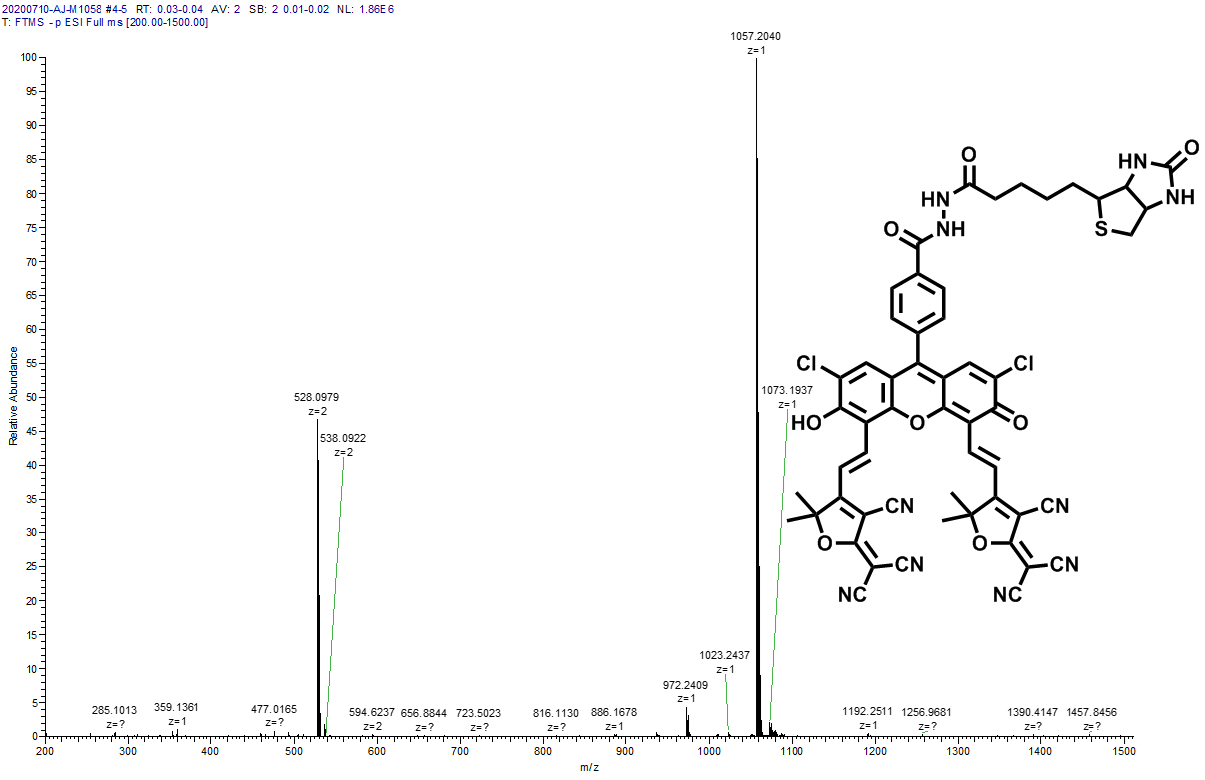


**Supplementary Figure 11.** HRMS of compound **1**.


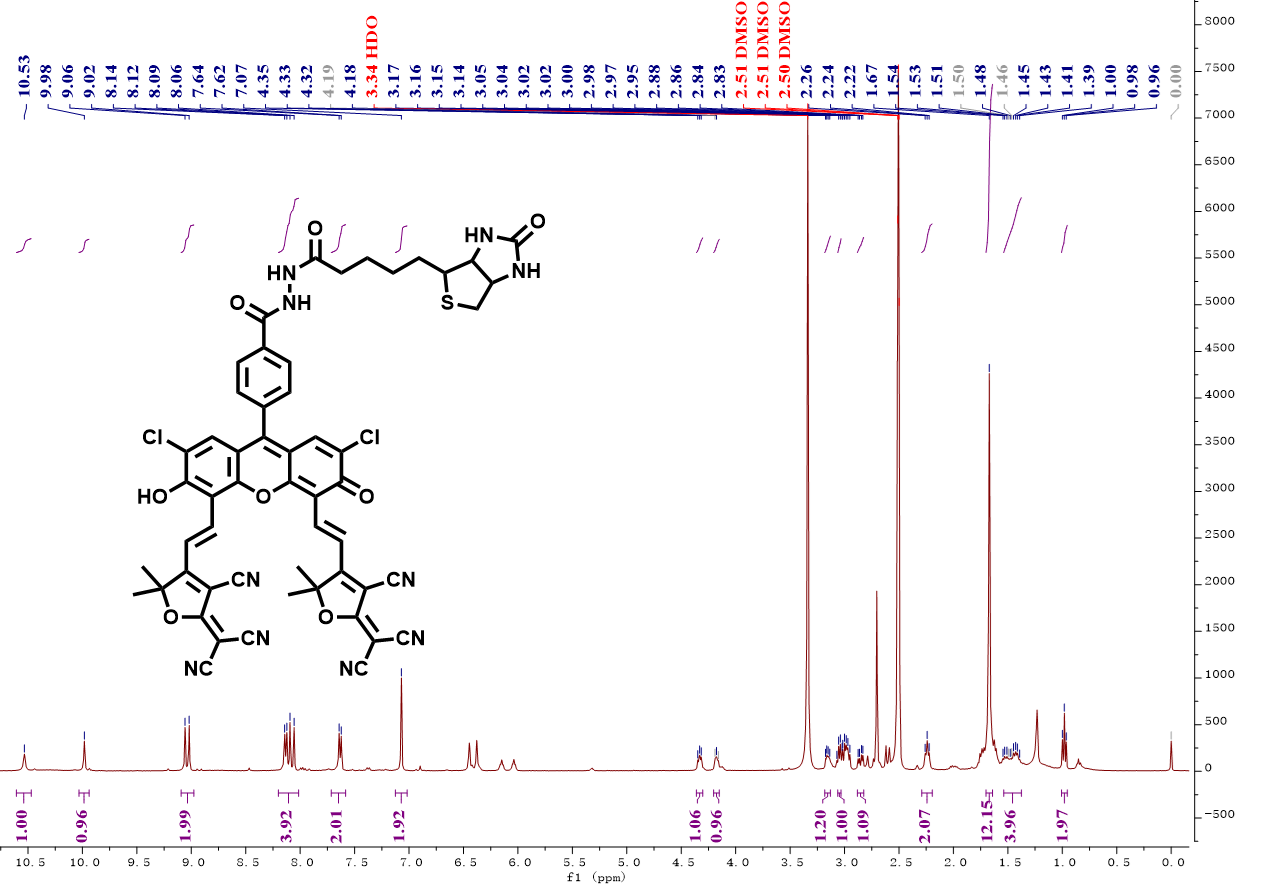


**Supplementary Figure 12.** ^1^H-NMR spectrum of compound **1** in (CD_3_)_2_SO.


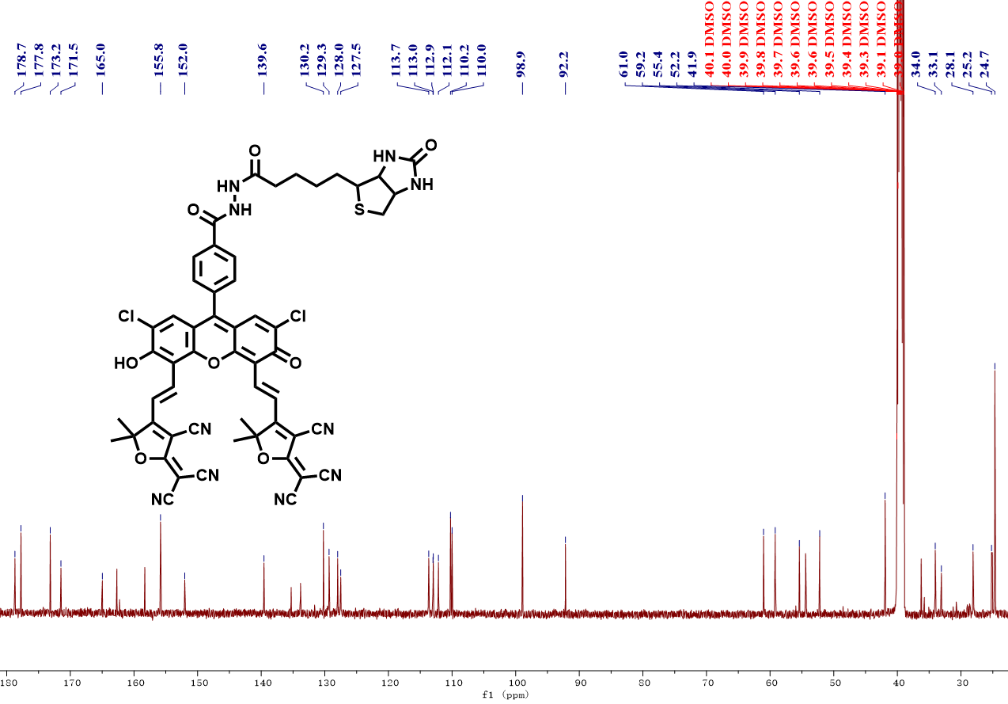


**Supplementary Figure 13.** ^13^C-NMR spectrum of compound **1** in (CD_3_)_2_SO.


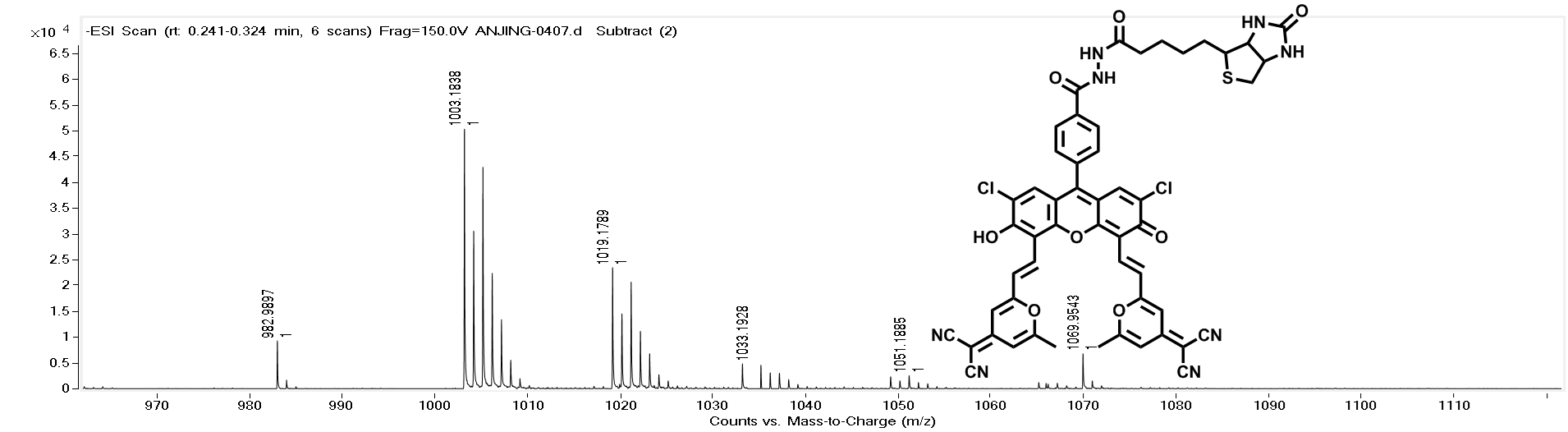


**Supplementary Figure 14.** HRMS of compound **2**.


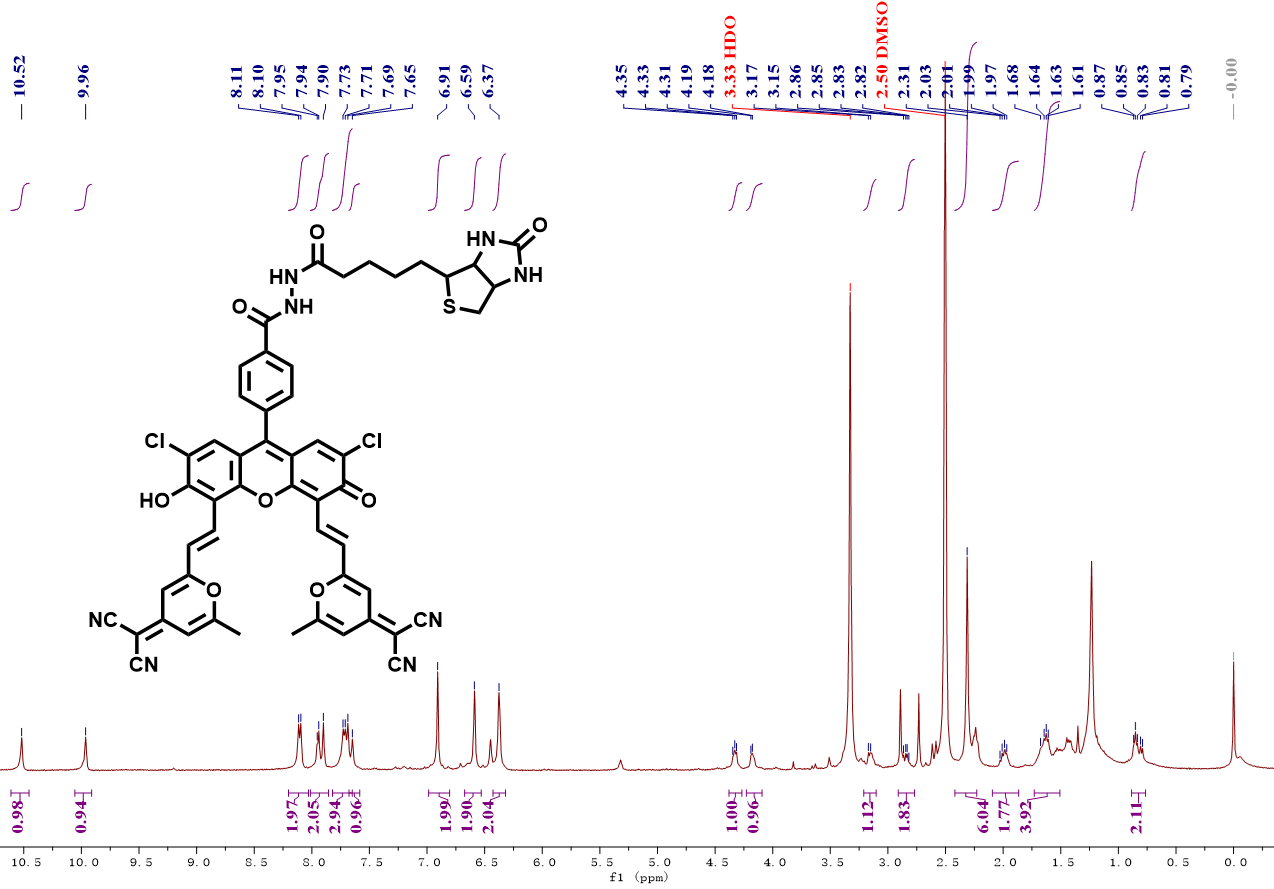


**Supplementary Figure 15.** ^1^H-NMR spectrum of compound **2** in (CD_3_)_2_SO.


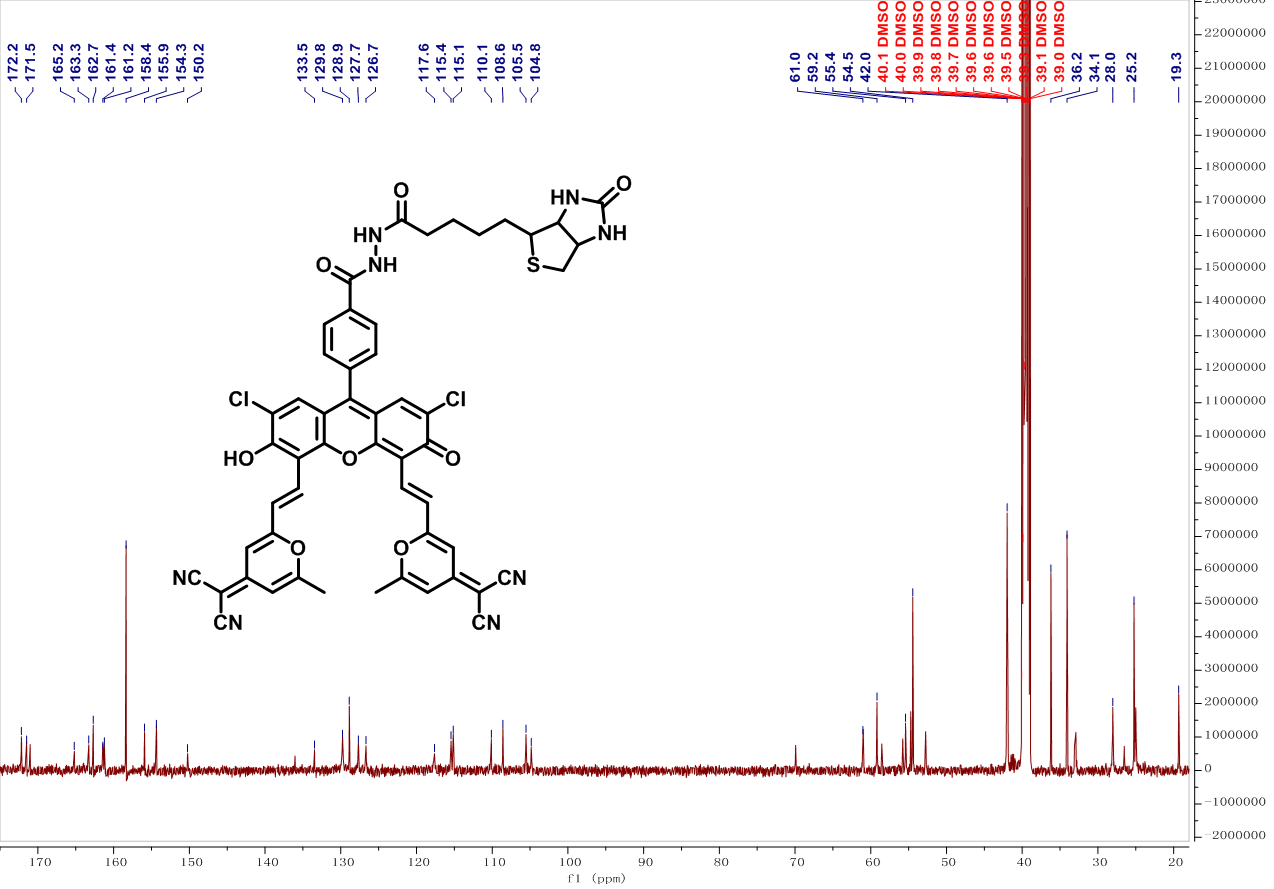


**Supplementary Figure 16.** ^13^C-NMR spectrum of compound **2** in (CD_3_)_2_SO.


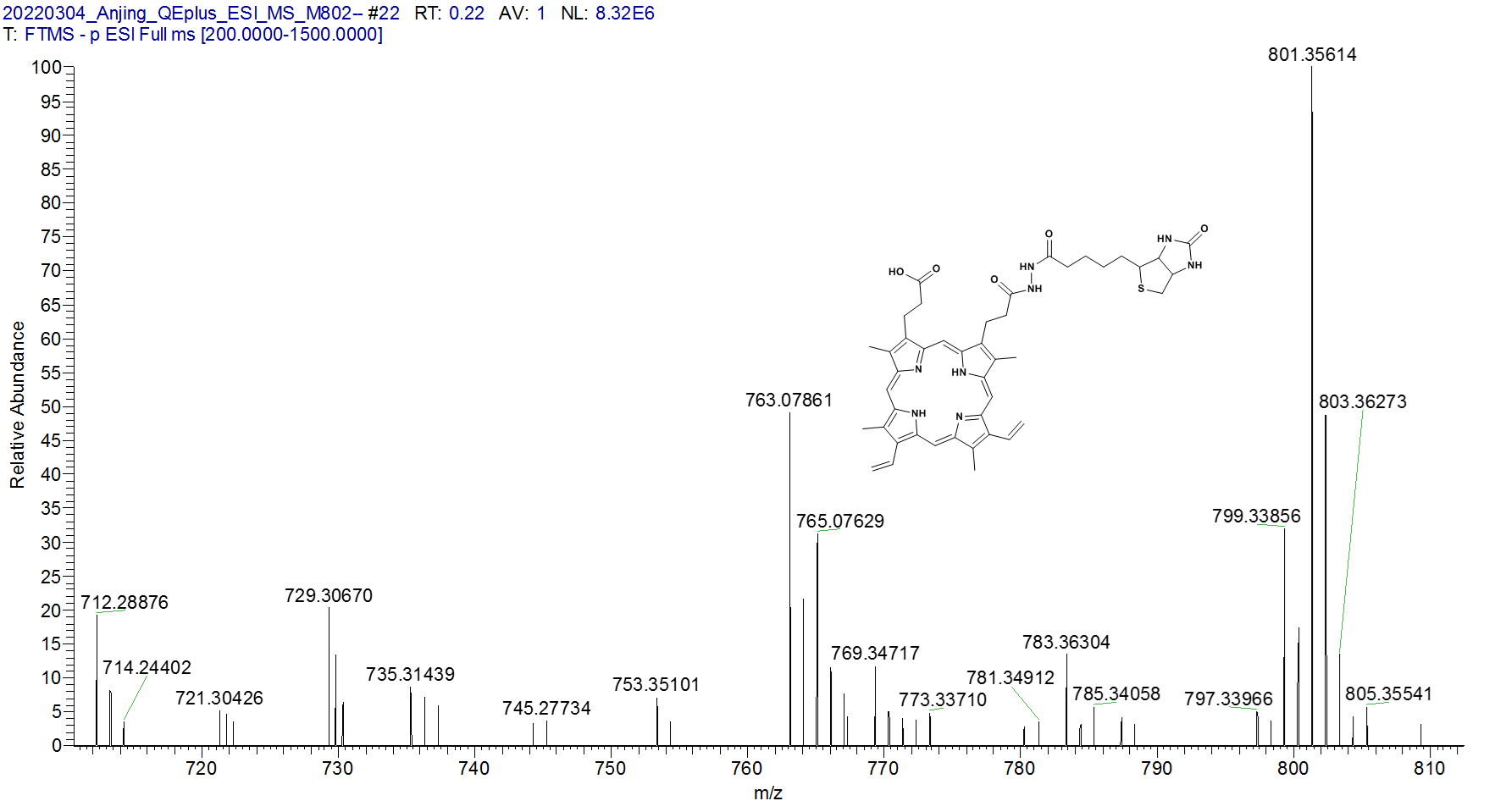


**Supplementary Figure 17.** HRMS of compound **3**.


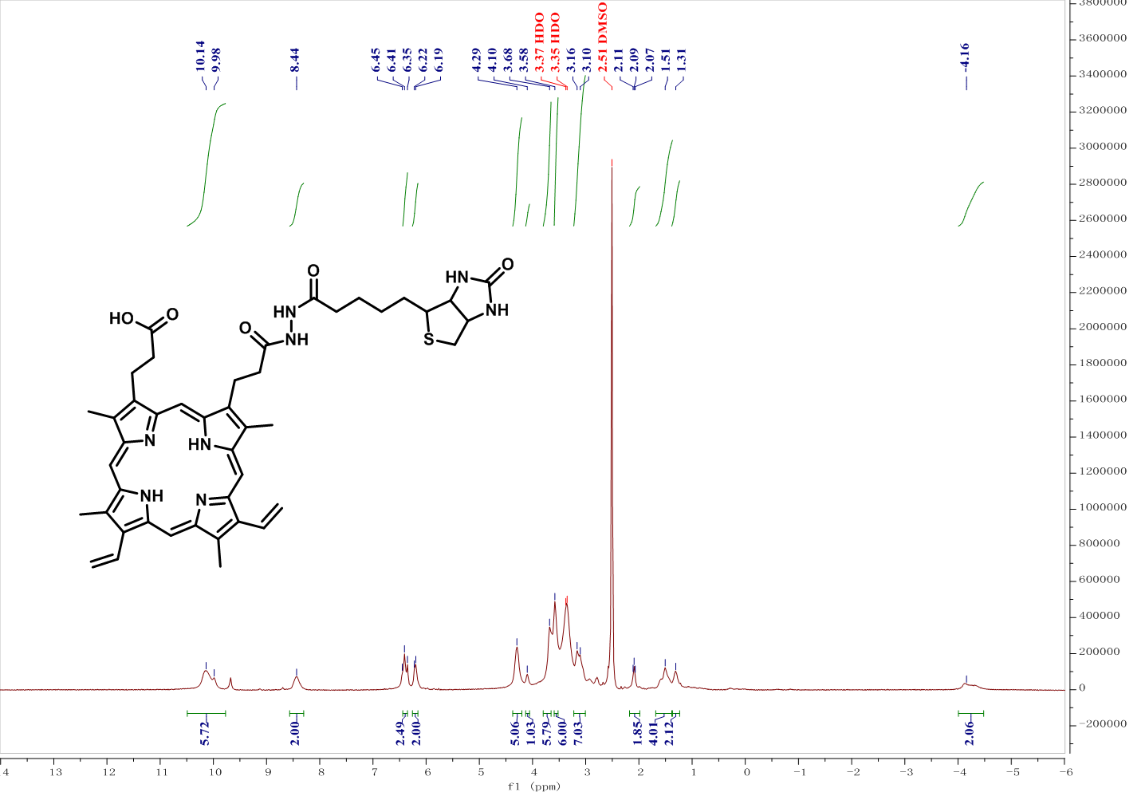


**Supplementary Figure 18.** ^1^H-NMR spectrum of compound **3** in (CD_3_)_2_SO.


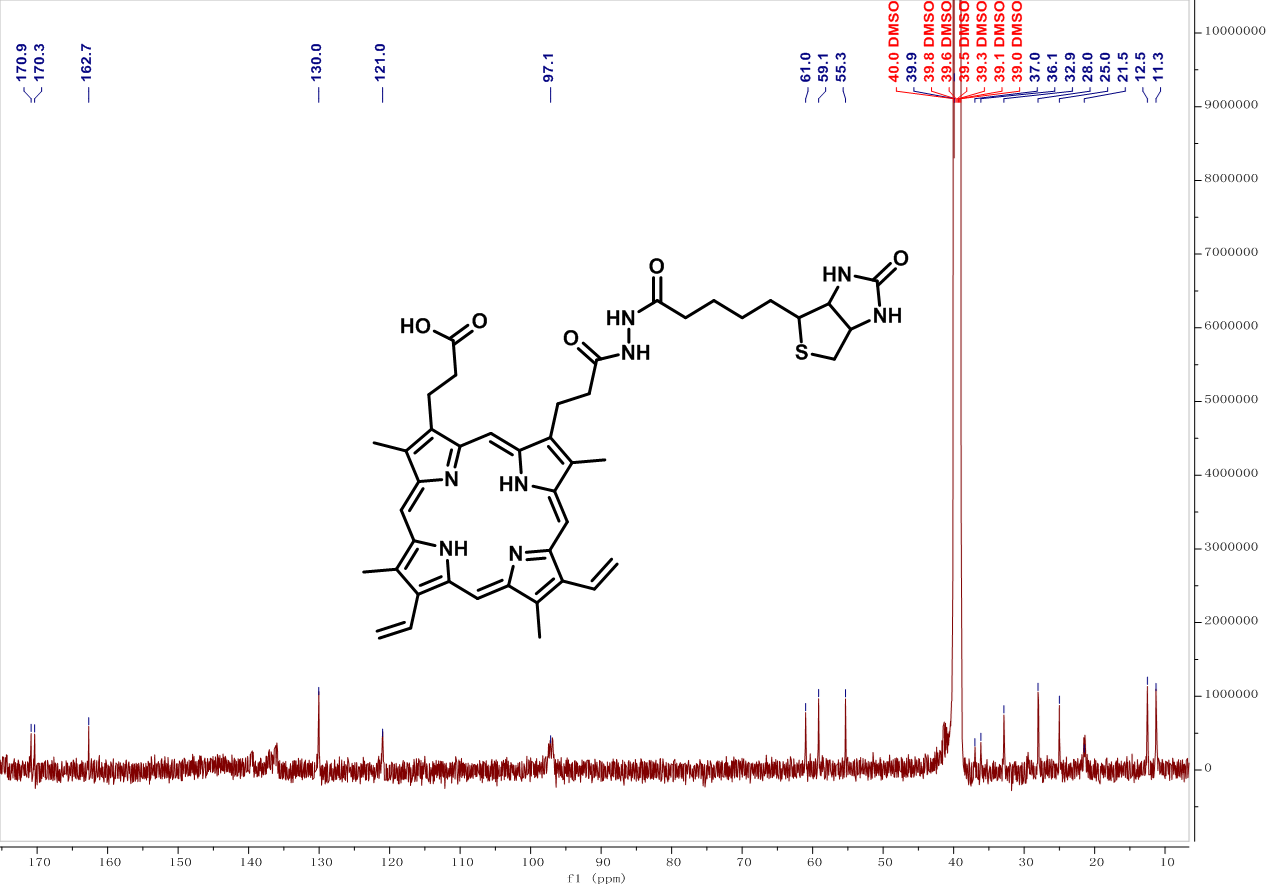


**Supplementary Figure 19.** ^13^C-NMR spectrum of compound **3** in (CD_3_)_2_SO.





**Supplementary Figure 20.** FTIR of compound **1,4** and **11**.





**Supplementary Figure 21.** FTIR of compound **2** and **5**.





**Supplementary Figure 22.** FTIR of compound **3**.





**Supplementary Figure 23.** Absorption spectra of compound **1** (10 μM) and emission spectra of white light LED.


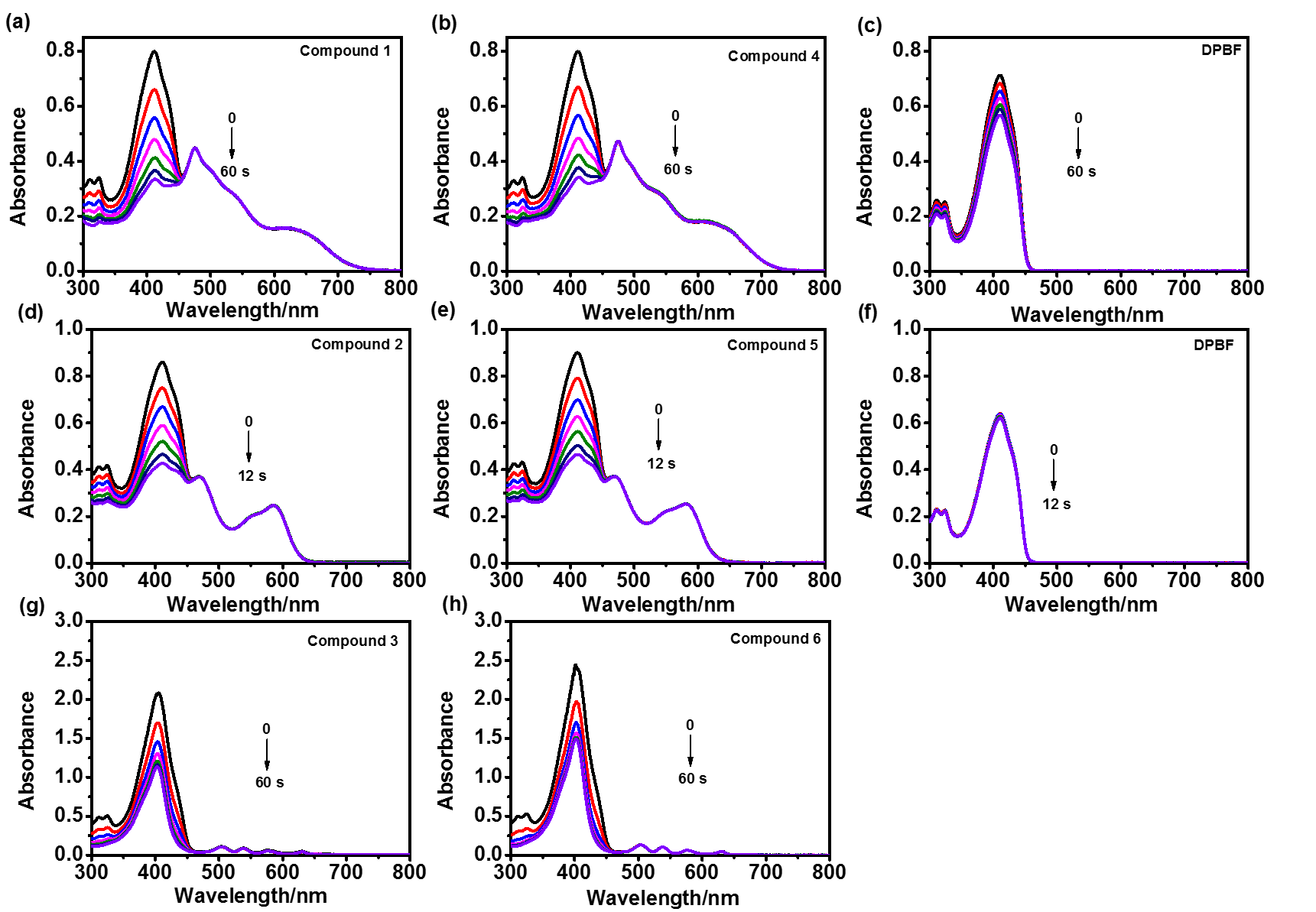


**Supplementary Figure 24.** UV−vis spectra of DPBF (50 μM) in the presence of (a) compound **1**, (b) **4**, (d) **2**, (e) **5**, (g) **3**, (h) **6** or (c), (f) in ethanol under white light irradiation (20 mW cm^−2^).


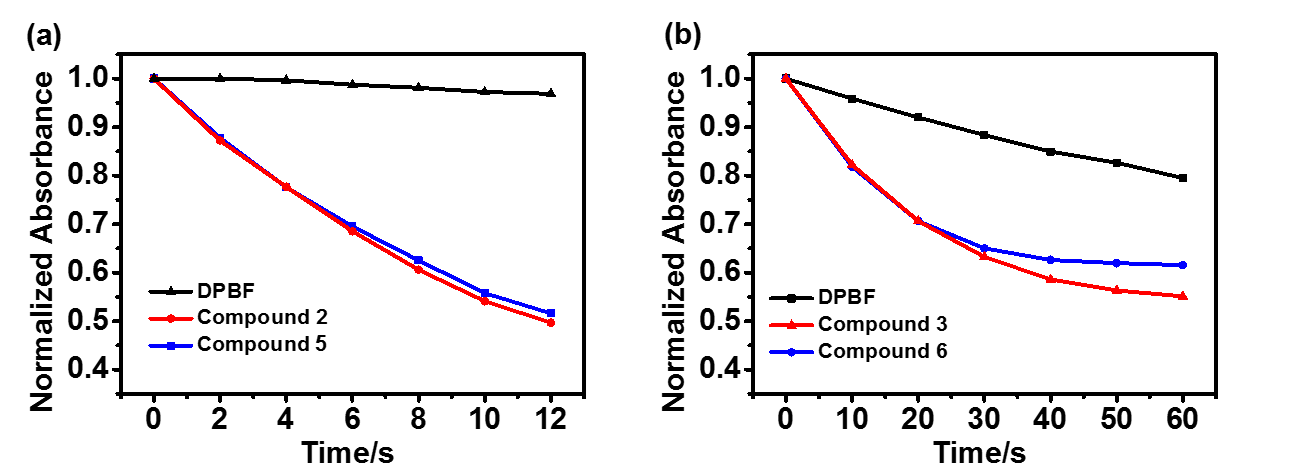


**Supplementary Figure 25.** Normalized absorbance of DPBF at 411 nm in the presence of photosensitizers as a function of irradiation time.


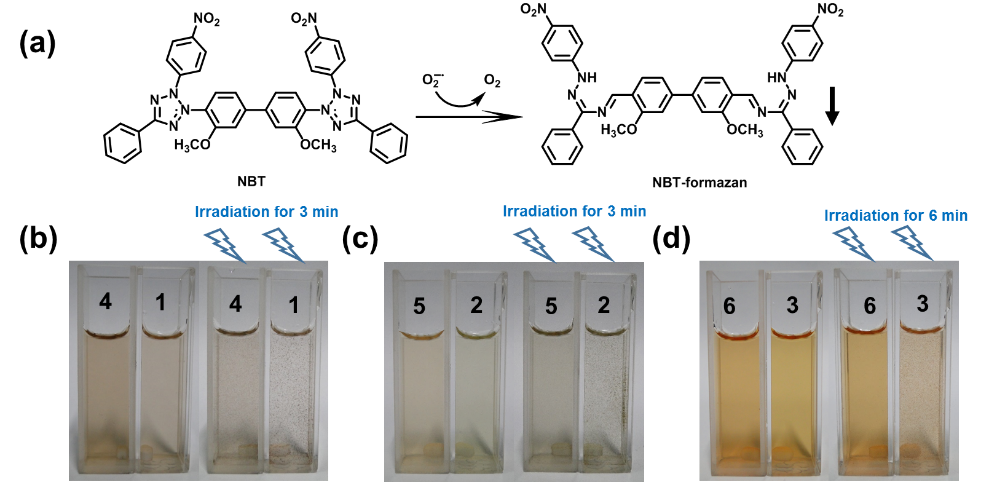


**Supplementary Figure 26.** (a) Schematic illustration of NBT for O_2_^−•^ detection. (b) O_2_^−•^ generation of the mixture solution of NBT with compound **4** or **1** before (left) and after (right) white light irrdiation (20 mW/cm^2^, 3 min). (c) O_2_^−•^ generation of the mixture solution of NBT with compound **5** or **2** before (left) and after (right) white light irradiation (20 mW/cm^2^, 3 min). (d) O_2_^−•^ generation of the mixture solution of NBT with compound **6** or **3** before (left) and after (right) white light irradiation (20 mW/cm^2^, 6 min).

**
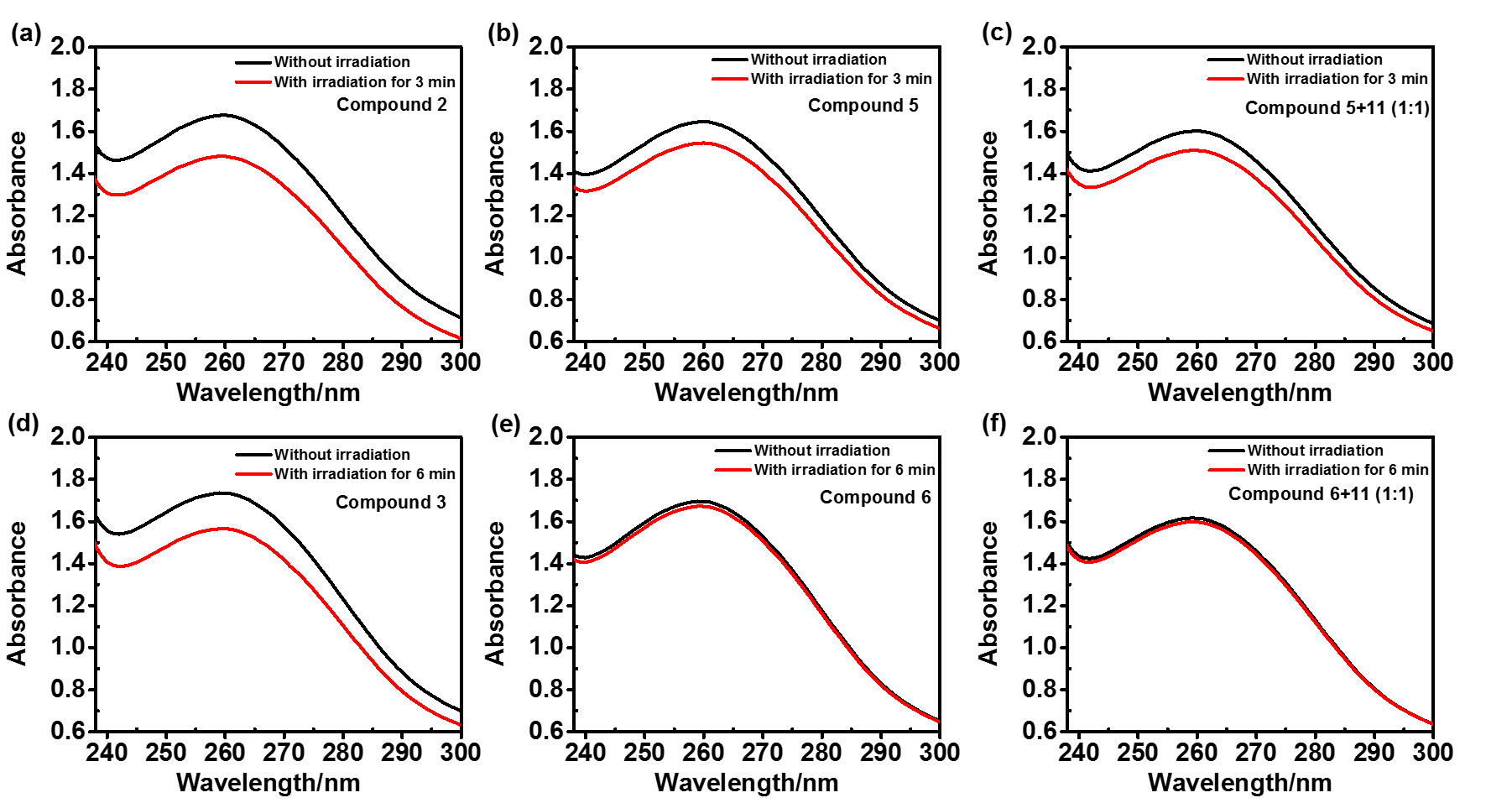
**

**Supplementary Figure 27.** Degradation of NBT by O_2_^−•^ under different treatments.


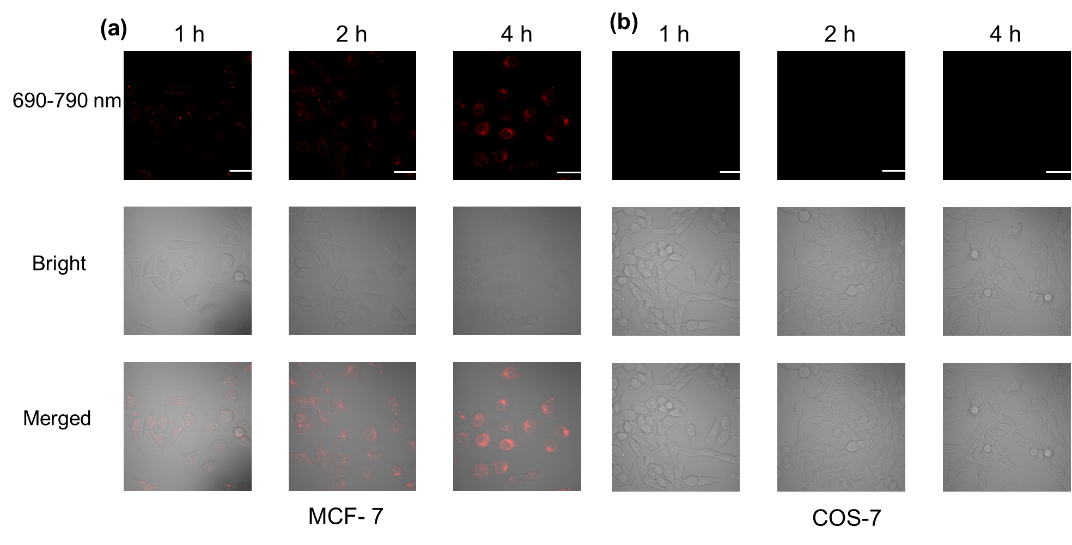


**Supplementary Figure 28.** Confocal images of MCF-7 cells and COS-7 cells with compound **1** (10 μM) at different incubation times. Scale bars :30 μm. The experiment was repeated three times independently, with similar results.


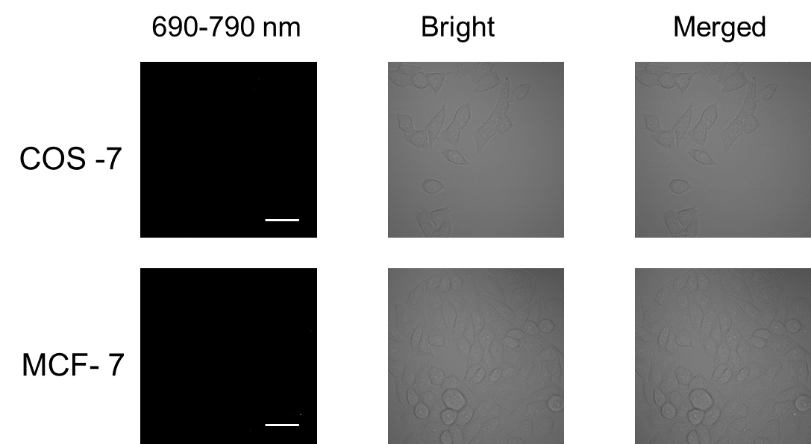


**Supplementary Figure 29.** Confocal images of COS-7 cells and MCF-7 cells with compound **4** (10 μM) incubated for 4 h, respectively. Scale bars :30 μm. The experiment was repeated three times independently, with similar results.





**Supplementary Figure 30.** Cell viability of MCF-7 cells treated with increasing concentrations of compound **1** in the dark. Data are presented as mean ± s.d. from four independent replicates.





**Supplementary Figure 31.** Dark toxicity and photocytotoxicity of compound **3** and **6** on MCF-7 cells (a)under normoxia (21% O_2_) and (b) hypoxia (1% O_2_) (white light 20 mW/cm^2^, 5min). Data are presented in (a) and (b) as mean ± s.d. from six independent replicates.


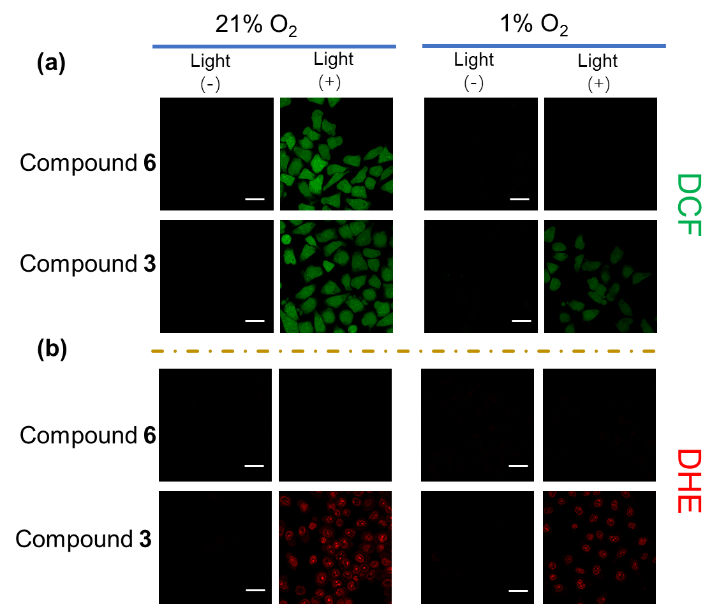


**Supplementary Figure 32.** Confocal fluorescence images of (a) ROS and (b) O_2_^−•^ generation in cells incubated with compound **3** and **6**, respectively. (“+” represents with irradiation, “-” represents without irradiation) Scale bars :30 μm. The experiment was repeated three times independently, with similar results.


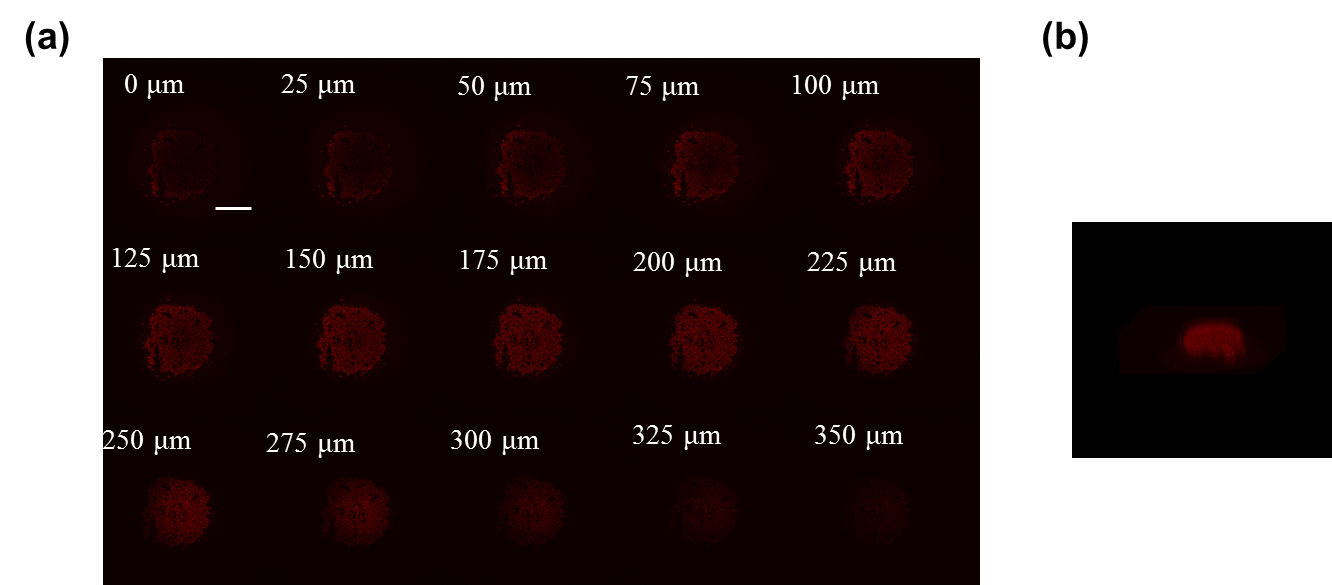


**Supplementary Figure 33.** Z-stack images after incubation of (a)compound **1** (10 μM) for 4 h in MCF-7 cells MCTS, respectively. Z-axis images scanning from the top to the bottom of an intact spheroid every 25 μm. (b) 3D Z-stack of an intact spheroid. Scale bars :400 μm. The experiment was repeated three times independently, with similar results.





**Supplementary Figure 34.** Photostability of compound **1** (10 μM) in cell culture medium, which was exposed to a white light (400-800 nm, 40 mW/cm^2^). Fluorescence intensity values of 733 nm for compound **1** was recorded after every 20 min. The excitation wavelength was 515 nm.


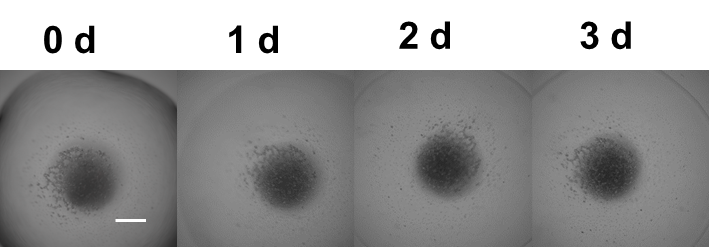


**Supplementary Figure 35.** Photocytotoxicity effects of white light irradiation on MCF-7 cells spheroids (40 mW/cm^2^, 20 min). Scale bars :400 μm. The experiment was repeated three times independently, with similar results.


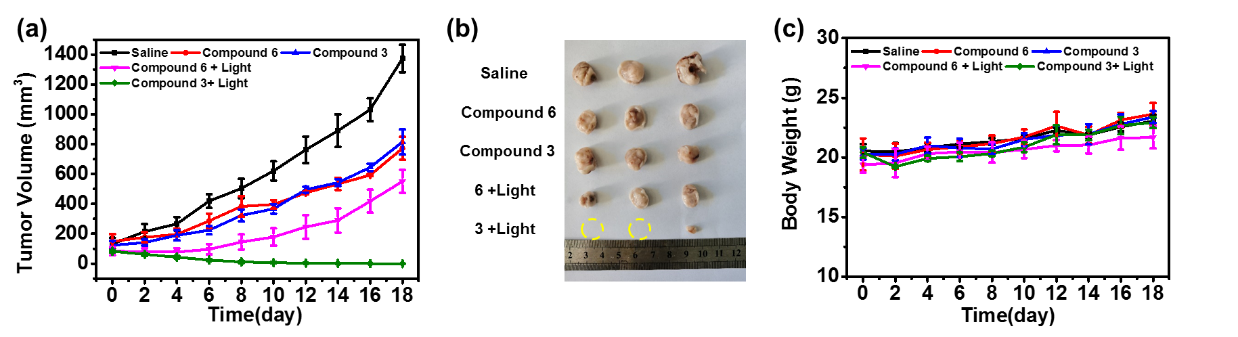


**Supplementary Figure 36.** (a) Tumor volume of each group of 4T1 tumor bearing mice during the treatment period. PpIX or PpIX-Biotin (100 μmol/mL, 100 μL) was intravenously injected. Tumor volume was calculated as (width)^2^ × (length) × 0.5. (b)Representative pictures for tumors excised from the mice with different treatments after 18 day of treatment. (c)Body weights of mice in different groups during treatment. Data are presented in (a) and (c) as mean ± s.d. from three independent replicates.

**Supplementary Table 1.** Cartesian coordinates of optimized compound **1** calculated by the DFT, B3LYP/6-31G(d), Gaussian 09 program.

| C | 5.74495 | -1.56823 | -2.27062 |
| --- | --- | --- | --- |
| C | 4.35839 | -1.63738 | -2.20635 |
| C | 3.71905 | -1.79903 | -0.96772 |
| C | 4.49205 | -1.96199 | 0.191 |
| C | 5.87885 | -1.89438 | 0.12327 |
| C | 6.51432 | -1.67413 | -1.10542 |
| C | 2.24879 | -1.70318 | -0.83194 |
| C | 1.50483 | -2.80115 | -0.28075 |
| C | 0.11376 | -2.66362 | -0.1071 |
| O | -0.48422 | -1.47397 | -0.34492 |
| C | 0.1881 | -0.40459 | -0.84725 |
| C | 1.57631 | -0.5329 | -1.17227 |
| C | -0.54312 | 0.76735 | -0.99231 |
| C | 0.06884 | 1.89762 | -1.70881 |
| C | 1.49099 | 1.72097 | -2.06728 |
| C | 2.20996 | 0.615 | -1.76925 |
| C | 2.0895 | -4.04761 | 0.03694 |
| C | 1.31531 | -5.07358 | 0.52495 |
| C | -0.08428 | -4.93414 | 0.67174 |
| C | -0.71833 | -3.71745 | 0.32049 |
| Cl | 2.2487 | 3.06808 | -2.8801 |
| O | -0.53232 | 2.9482 | -1.95772 |
| C | -2.16478 | -3.65716 | 0.41792 |
| C | -1.82922 | 0.89493 | -0.35348 |
| Cl | 2.04261 | -6.60867 | 0.94567 |
| O | -0.86268 | -5.93133 | 1.10338 |
| C | -2.99737 | -2.80605 | -0.23882 |
| C | -2.80816 | 1.78197 | -0.689 |
| C | -4.41925 | -2.79502 | -0.10368 |
| C | -4.01989 | 1.98615 | 0.03357 |
| C | -5.25407 | -1.93069 | -0.77875 |
| C | -6.60582 | -2.12659 | -0.30476 |
| O | -6.63554 | -3.13783 | 0.55986 |
| C | -5.27649 | -3.67129 | 0.79066 |
| C | -4.28171 | 1.70473 | 1.50289 |
| O | -5.64544 | 2.23473 | 1.69929 |
| C | -6.12815 | 2.72984 | 0.55979 |
| C | -5.13644 | 2.6157 | -0.47954 |
| C | -4.36954 | 0.23456 | 1.90392 |
| C | -5.01293 | -3.50626 | 2.2873 |
| C | -5.30343 | -5.13269 | 0.33882 |
| C | -3.34606 | 2.51049 | 2.40397 |
| C | -7.7584 | -1.4146 | -0.57635 |
| C | -7.41302 | 3.24205 | 0.5337 |
| C | 7.97578 | -1.37513 | -1.20125 |
| C | 2.23933 | 3.51379 | 0.93805 |
| C | 2.04547 | 2.11739 | 1.56529 |
| S | 3.62154 | 1.18195 | 1.36939 |
| C | 4.61149 | 2.73305 | 1.24597 |
| C | 3.7002 | 3.67905 | 0.43984 |
| N | 3.94108 | 5.09363 | 0.68172 |
| C | 3.09171 | 5.58598 | 1.65721 |
| N | 2.14892 | 4.60332 | 1.9066 |
| O | 3.16059 | 6.68454 | 2.19734 |
| C | 5.97087 | 2.5043 | 0.5885 |
| C | 6.92128 | 1.61493 | 1.39525 |
| C | 8.26198 | 1.40252 | 0.68543 |
| C | 9.19508 | 0.45372 | 1.47254 |
| C | 10.30022 | -0.11504 | 0.60281 |
| N | 10.04301 | -1.34029 | -0.00113 |
| O | 11.38716 | 0.42278 | 0.44005 |
| N | 8.7445 | -1.7901 | -0.14068 |
| O | 8.44845 | -0.71087 | -2.11909 |
| C | -4.84417 | -1.01218 | -1.7778 |
| N | -4.46183 | -0.29151 | -2.60707 |
| C | -5.25939 | 3.0687 | -1.81612 |
| N | -5.30059 | 3.43629 | -2.91943 |
| C | -7.98895 | 3.80717 | -0.63385 |
| N | -8.49673 | 4.29123 | -1.56425 |
| C | -8.20438 | 3.21574 | 1.71411 |
| N | -8.86094 | 3.19546 | 2.67665 |
| C | -8.9816 | -1.78382 | 0.04789 |
| N | -9.98724 | -2.07986 | 0.55559 |
| C | -7.76039 | -0.27169 | -1.41925 |
| N | -7.79076 | 0.67923 | -2.09167 |
| H | 6.24437 | -1.39889 | -3.21763 |
| H | 3.76741 | -1.54682 | -3.11164 |
| H | 4.00613 | -2.07416 | 1.15363 |
| H | 6.44699 | -1.95516 | 1.04418 |
| H | 3.26473 | 0.5712 | -1.99895 |
| H | 3.15092 | -4.19784 | -0.10889 |
| H | -2.58865 | -4.42684 | 1.04971 |
| H | -1.99553 | 0.20868 | 0.46965 |
| H | -0.3224 | -6.71514 | 1.31947 |
| H | -2.59308 | -2.07149 | -0.92363 |
| H | -2.6889 | 2.36863 | -1.59085 |
| H | -4.70759 | 0.174 | 2.94166 |
| H | -5.08789 | -0.2833 | 1.26926 |
| H | -3.40128 | -0.26294 | 1.83203 |
| H | -4.03155 | -3.90357 | 2.55269 |
| H | -5.0545 | -2.4542 | 2.57696 |
| H | -5.77355 | -4.05785 | 2.84526 |
| H | -6.06472 | -5.66802 | 0.91191 |
| H | -5.54066 | -5.20064 | -0.72579 |
| H | -4.33678 | -5.61025 | 0.51353 |
| H | -2.32318 | 2.1413 | 2.29323 |
| H | -3.36747 | 3.56918 | 2.13312 |
| H | -3.65392 | 2.39818 | 3.44669 |
| H | 1.53011 | 3.66463 | 0.1161 |
| H | 1.25258 | 1.55382 | 1.07173 |
| H | 1.79679 | 2.21565 | 2.62414 |
| H | 4.74455 | 3.14125 | 2.25467 |
| H | 3.78451 | 3.43824 | -0.62177 |
| H | 4.8736 | 5.48182 | 0.62592 |
| H | 1.24702 | 4.90181 | 2.25407 |
| H | 5.81504 | 2.07001 | -0.40825 |
| H | 6.44132 | 3.48579 | 0.4349 |
| H | 7.08582 | 2.05332 | 2.38869 |
| H | 6.44964 | 0.63726 | 1.56037 |
| H | 8.07371 | 1.00325 | -0.31582 |
| H | 8.77013 | 2.36304 | 0.53781 |
| H | 9.67744 | 0.99704 | 2.28968 |
| H | 8.60934 | -0.36016 | 1.90826 |
| H | 10.68631 | -1.60442 | -0.74201 |
| H | 8.46393 | -2.55608 | 0.45682 |
