# Supplementary material for: An unexpected strategy to alleviate hypoxia limitation of photodynamic therapy by biotinylation of photosensitizers
Source: Nat Commun. 2022 Apr 25;13:2225. doi: 10.1038/s41467-022-29862-9 (PMC9038921; doi:10.1038/s41467-022-29862-9)
Supplement: Supplementary file 3 — Reporting Summary [file 41467_2022_29862_MOESM3_ESM.pdf]

nature portfolio

Corresponding author(s): Fengling Song
Last updated by author(s): Mar 21, 2022

Reporting Summary

Nature Portfolio wishes to improve the reproducibility of the work that we publish. This form provides structure for consistency and transparency in reporting. For further information on Nature Portfolio policies, see our Editorial Policies and the Editorial Policy Checklist.

Statistics

For all statistical analyses, confirm that the following items are present in the figure legend, table legend, main text, or Methods section.

n/a	Confirmed	
☐	☒	The exact sample size (n) for each experimental group/condition, given as a discrete number and unit of measurement
☐	☒	A statement on whether measurements were taken from distinct samples or whether the same sample was measured repeatedly
☒	☐	The statistical test(s) used AND whether they are one- or two-sided<br>*Only common tests should be described solely by name; describe more complex techniques in the Methods section.*
☒	☐	A description of all covariates tested
☒	☐	A description of any assumptions or corrections, such as tests of normality and adjustment for multiple comparisons
☐	☒	A full description of the statistical parameters including central tendency (e.g. means) or other basic estimates (e.g. regression coefficient) AND variation (e.g. standard deviation) or associated estimates of uncertainty (e.g. confidence intervals)
☒	☐	For null hypothesis testing, the test statistic (e.g. F, t, r) with confidence intervals, effect sizes, degrees of freedom and P value noted<br>*Give P values as exact values whenever suitable.*
☒	☐	For Bayesian analysis, information on the choice of priors and Markov chain Monte Carlo settings
☒	☐	For hierarchical and complex designs, identification of the appropriate level for tests and full reporting of outcomes
☐	☒	Estimates of effect sizes (e.g. Cohen's d, Pearson's r), indicating how they were calculated

Our web collection on statistics for biologists contains articles on many of the points above.

Software and code

Policy information about availability of computer code

Data collection	Confocal laser scanning microscopy images were acquired on Olympus FV-1000; Absorbance and fluorescence spectra were recorded on UV –vis spectrophotometer (Agilent Tech) and fluorometer (Agilent Tech); MTT assays were measured on Microplate reader named Multiskan FC from Thermo scientific. Cyclic voltammetry measurement was conducted by using CHI760E Electrochemical workstation. Optimized compound 1 was calculated by the DFT, B3LYP/6-31G(d), Gaussian 09 program.
Data analysis	OriginPro 9 and Image J bundled with 64-bit Java 1.8.0_172 and FV10 -ASW 3.0 Viewer.

For manuscripts utilizing custom algorithms or software that are central to the research but not yet described in published literature, software must be made available to editors and reviewers. We strongly encourage code deposition in a community repository (e.g. GitHub). See the Nature Portfolio guidelines for submitting code & software for further information.

Data

Policy information about availability of data

All manuscripts must include a data availability statement. This statement should provide the following information, where applicable:

- Accession codes, unique identifiers, or web links for publicly available datasets
- A description of any restrictions on data availability
- For clinical datasets or third party data, please ensure that the statement adheres to our policy

The experimental data generated in this study are available within the article, Supplementary Information, and Source data.The raw data underlying Fig. 2,3,4,7 and Supplementary Figs.20,21,22, 23,24,25,27,30,31,34 and 36 are provided as a Source data file.

Field-specific reporting

Please select the one below that is the best fit for your research. If you are not sure, read the appropriate sections before making your selection.

☒ Life sciences ☐ Behavioural & social sciences ☐ Ecological, evolutionary & environmental sciences

For a reference copy of the document with all sections, see nature.com/documents/nr-reporting-summary-flat.pdf

Life sciences study design

All studies must disclose on these points even when the disclosure is negative.

Sample size	Sample sizes were chosen based on the minimum number of animals/replicates to support meaningful conclusions, and the sample size (n) of each experiment is provided in the corresponding Figure legends in the paper and supplementary information.
Data exclusions	No data were excluded.
Replication	Cell and animal experiments were replicated using samples from 3-6 independent biological samples. Exact numbers for each individual type of experiment are provided in Figure legends and in the Source Data file. All attempts at replicating results were successful.
Randomization	Fifteen mice were randomly divided into five groups. The cells and animal used in this paper were randomly distributed into different groups for experiments when the cells were in logarithmic growth or tumor volumes reached the proper size (about 100 mm3).The cells were randomly seeded in a cell-culture plates.
Blinding	The technician collected and analyzed data without knowing the group assignment for each sample..

Reporting for specific materials, systems and methods

We require information from authors about some types of materials, experimental systems and methods used in many studies. Here, indicate whether each material, system or method listed is relevant to your study. If you are not sure if a list item applies to your research, read the appropriate section before selecting a response.

Materials & experimental systems

n/a	Involved in the study	
☒	☐	Antibodies
☐	☒	Eukaryotic cell lines
☒	☐	Palaeontology and archaeology
☐	☒	Animals and other organisms
☒	☐	Human research participants
☒	☐	Clinical data
☒	☐	Dual use research of concern

Methods

n/a	Involved in the study	
☒	☐	ChIP-seq
☒	☐	Flow cytometry
☒	☐	MRI-based neuroimaging

Eukaryotic cell lines

Policy information about cell lines

Cell line source(s)	MCF-7 cells ,COS-7 cells and 4T1 cells were purchased from Institute of Basic Medical Sciences (IBMS) of the Chinese Academy of Medical Sciences.
Authentication	None of cell lines used were authenticated.
Mycoplasma contamination	The cell lines tested negative for Mycoplasma.
Commonly misidentified lines (See ICLAC register)	No misidentified cell lines were used.

Animals and other organisms

Policy information about studies involving animals; ARRIVE guidelines recommended for reporting animal research

Laboratory animals	6-week-old female BALB/c mice .Mice were kept in transparent, non-toxic plastic boxes with stainless steel wire cages. Laminar flow cabinet and separate ventilation cage are used to purify the air in the cage. Mice were reared at 23-26 ?, with relative humidity of 50%-55% and alternating light and dark for 12h/12h.

Wild animals	No wild animals were used in the study.
Field-collected samples	No field collected samples were used in the study.
Ethics oversight	All animal experiments involved in this study have been approved by the local research ethics review board of the Animal Ethics Committee of Dalian University of Technology (Certificate number//Ethics approval no. is 2018–043).

Note that full information on the approval of the study protocol must also be provided in the manuscript.
